# Factors influencing college students’ willingness to participate in sports-based disability assistance volunteer services

**DOI:** 10.3389/fpsyg.2026.1728609

**Published:** 2026-01-21

**Authors:** Yunxiang Lin, Lingyan Yan, Zifeng Shen

**Affiliations:** 1 School of Physical Education and Sport Science, Fujian Normal University, Fuzhou, Fujian, China; 2 School of Communication, Fujian Normal University, Fuzhou, Fujian, China; 3 School of Psychology, Fujian Normal University, Fuzhou, Fujian, China

**Keywords:** college students, motivational mechanism, sports-based disability support volunteer services, theory of planned behavior, willingness to participate

## Abstract

Sports-based disability assistance volunteer services play a crucial role in promoting social inclusion and harmonious development, with college students serving as the primary participant group in such initiatives. To explore the underlying mechanisms driving university students’ participation in these volunteer services, this study constructs an extended Theory of Planned Behavior model. Building upon the traditional constructs of the Theory of Planned Behavior (Behavioral Attitude, Subjective Norms, Perceptual-Behavioral Control, and Willingness to Participate), this model introduces the core variable Level of Awareness. Data analysis was conducted using structural equation modeling and mediation analysis based on questionnaires collected from 697 college students in China. The structural model demonstrated good fit. Key findings are as follows: The SEM model fit well: RMSEA = 0.06, CFI = 0.95. Level of Awareness significantly and directly influenced Willingness to Participate, while also significantly and positively predicting Behavioral Attitude, Subjective Norms, and Perceptual-Behavioral Control. Concurrently, Behavioral Attitude (*β* = 0.31, *p* < 0.001), Subjective Norms (*β* = 0.30, *p* < 0.01), and Perceptual-Behavioral Control (*β* = 0.25, *p* < 0.01) significantly predicted Willingness to Participate, partially mediating this relationship. This study confirms that Level of Awareness is a key antecedent variable for stimulating behavioral intention, providing new theoretical perspectives and practical insights for recruiting and mobilizing youth volunteers in Chinese universities or official social organizations: (1) Factors that influence the level of awareness of sports programs for people with disabilities significantly affect the intention to participate; higher levels of awareness are associated with stronger intentions to participate. (2) The level of awareness, as a core factor, positively influences behavioral attitude, subjective norms, perceptual-behavioral control, and willingness to participate, and therefore constitutes the core of the theoretical model. (3) Behavioral attitude, subjective norms, and perceptual-behavioral control each significantly influence willingness to participate; the path coefficients for behavioral attitude and subjective norms are slightly larger than the path coefficient for perceptual-behavioral control. These three variables mediate the relationship between the level of awareness and intention to participate in sports-based volunteer services for people with disabilities.

## 1 Introduction

### 1.1 Research background

The *Healthy China 2030* Planning Outline proposes strengthening health services for key populations and continuously meeting the growing rehabilitation and fitness needs of persons with disabilities. According to data released by the National Bureau of Statistics of China, by the end of 2023, the total number of persons with disabilities in China reached approximately 85.914 million, accounting for 6.16% of the total population and showing an upward trend (Ramlan et al., 2023). Meeting the rehabilitation needs of this large population is a priority for contemporary society. Physical exercise is a vital pathway for persons with disabilities to achieve health, and they urgently require professional rehabilitation guidance to access high-quality sports and fitness services. Sports assistance for persons with disabilities is one of the key activities that help individuals with disabilities complete rehabilitation exercises. It also provides them with additional social channels, boosting their self-confidence and sense of well-being (Aitchison et al., 2022). However, sports assistance for persons with disabilities relies heavily on public volunteer support and societal backing. Among these volunteers, university students serve as the “main force” in such initiatives, playing a vital role in promoting the widespread adoption and sustainable development of sports activities for people with disabilities. Conversely, this involvement also strengthens the sense of social responsibility and civic awareness among the student population.

Existing research primarily focuses on the challenges that people with disabilities encounter when participating in public sports activities and proposes solutions from multiple perspectives, including disability federations, disability-related initiatives, social sports instructors, and organizations responsible for allocating sports resources (Liang et al., 2022; Dianbo and Cheng, 2021; Jingyuan and Guoxiang, 2023). Research participants primarily included Paralympic athletes and organizations serving people with disabilities (Runji et al., 2020; Qing-hua et al., 2023; Jiangang, 2023). Additionally, the more frequently people with disabilities participate in sports activities, the greater their sense of physical and mental well-being (Hassett et al., 2024). However, professional support is essential during participation to ensure that people with disabilities acquire proper exercise techniques (Hill et al., 2024). Ramlan et al. (2023) found that individuals with disabilities in Malaysia have fewer opportunities to participate in outdoor activities, while local acceptance of their involvement in recreational pursuits remains at a low to moderate level. This underscores the critical importance of social acceptance in enabling persons with disabilities to engage in nature-based leisure activities (Ramlan et al., 2023).

Although these studies provide insight into rehabilitation exercises for people with disabilities, they focus solely on people with disabilities as subjects and lack sufficient theoretical explanation. Notably, university students’ perceptions of people with disabilities participating in physical exercise — and the psychological mechanisms that underlie those perceptions — have not been adequately elucidated. Cavusoglu et al. (2014) found that university students who had taken courses on disability held different attitudes toward people with disabilities than those who had not taken such courses. However, their study did not identify cognitive interventions that effectively changed students’ perceptions. Existing research also concentrates on college students’ intentions to engage in volunteer activities — such as programs for older adults and rural revitalization — but the psychological and behavioral mechanisms underlying students’ participation in sports-assistance volunteer programs for people with disabilities remain unexplored (Zhou and Chen, 2025; Chen et al., 2024; Hu et al., 2023).

To address these shortcomings, this study presents a scale measuring college students’ understanding of, and involvement in, sports-based disability-assistance programs. The Theory of Planned Behavior (TPB) will serve as the foundational framework and will be extended to examine the factors and mechanisms influencing university students’ participation in volunteer sports-assistance services for people with disabilities. This study examines the pathways by which students’ behavioral attitude toward the volunteer activities, their subjective norms, perceived-behavioral control, and willingness to participate influence actual participation.

### 1.2 Theoretical analysis and research hypotheses

**Figure 1:**
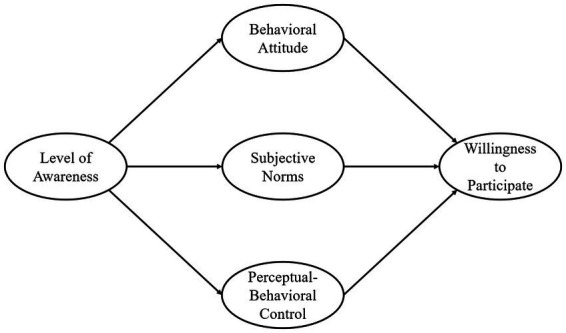
Theoretical analysis framework.

This study employs the Theory of Planned Behavior (TPB) as its primary theoretical framework. By integrating the proposed model with college students’ level of awareness regarding sports-based disability assistance, the study proposes a more comprehensive theoretical framework to examine students’ willingness to volunteer in related activities (see Figure 1). The Theory of Planned Behavior (TPB), proposed by Icek Ajzen, is a central theoretical framework for explaining behavioral intentions (Fishbein and Ajzen, 1977). The theory posits three core constructs — attitude toward the behavior, subjective norms, and perceived behavioral control — which together shape an individual’s behavioral intentions, the proximal predictor of actual behavior. Attitude refers to an individual’s overall positive or negative evaluation of performing a given behavior; subjective norms denote the perceived social pressure from significant others regarding whether one should engage in the behavior; and perceived behavioral control reflects an individual’s assessment of the ease or difficulty of performing the behavior, encompassing perceived self-efficacy as well as control over resources, external conditions, and personal capabilities. The TPB has been widely applied across fields such as sociology, health psychology, tourism studies, management, and environmental behavior research, where it has demonstrated strong explanatory and predictive power. In general, the more positive an individual’s attitude toward the behavior, the stronger the perceived normative pressure, and the lower the perceived difficulty, the stronger the individual’s behavioral intentions become; consequently, the likelihood of performing the behavior increases (Jinyu and Yuanbao, 2023).

This study introduces the domain-specific cognitive construct “level of awareness regarding sports-based disability assistance,” which refers to college students’ experiences and perceptions of volunteer activities in sports-based disability assistance. This concept differs significantly from attitudes, subjective norms, and perceptual-behavioral control within the Theory of Planned Behavior (TPB). Within the Theory of Planned Behavior (TPB), attitude refers to an individual’s positive or negative evaluation of performing a specific behavior. This evaluation is based on their belief about the outcomes that behavior will produce and their assessment of the value of those outcomes. Subjective norms refer to the perception of external social pressure, while perceived behavioral control denotes an individual’s sense of self-efficacy regarding their ability to perform certain behaviors. The level of awareness regarding sports-based disability volunteer services is primarily shaped by exposure to relevant policies and by the understanding and experience gained through participation in social practice. Traditional TPB theory has been validated in other disciplines and is applicable to research on volunteering behavior (Rongzi et al., 2023). Miller reviewed the literature on the TPB from 1996 to 2016 and reported that attitudes, subjective norms, and perceived behavioral control accounted for between 40 and 70% of the variance in behavioral intentions (Miller, 2017). The TPB demonstrates strong predictive power for many behavioral intentions. However, when used to explain complex volunteering behaviors, the original TPB framework may be insufficient and thus exhibits limitations.

To preserve the explanatory power of the TPB, the model should be extended. Accordingly, many studies adopt the traditional TPB as their theoretical foundation while incorporating additional variables. For example, Brayley et al. (2015) combined the Volunteer Function Inventory with the TPB to improve predictions of volunteering. Kao et al. (2019) used an expanded TPB to predict willingness to participate in science volunteering and found that satisfaction was the sole factor influencing sustained volunteering. A study of Chinese university students’ participation in volunteer services for the elderly found that adding personality-trait variables positively influenced intentions to continue volunteering (Chen et al., 2024). However, the theory of planned behavior (TPB) has seen limited application in sports volunteering research. Some scholars have demonstrated that college students’ perceptions of sports volunteering play a crucial role in shaping their willingness to participate (Dawei et al., 2023).

However, few studies have systematically integrated the level of awareness specific to sports-based disability assistance with the Theory of Planned Behavior (TPB) or explored its application pathways. Building on prior empirical findings, this study advances the Theory of Planned Behavior (TPB) by situating it within the specific context of sports-based disability assistance volunteering and extending it into an Extended Theory of Planned Behavior (ETPB) framework to enhance its behavioral predictive capacity. Specifically, the level of awareness of sports-based disability assistance volunteer services is conceptualized as a core antecedent variable. By integrating this construct into the TPB framework, the proposed model elucidates how college students’ cognitive understanding of such volunteer services systematically shapes their evaluations of behavioral outcomes (behavioral attitudes), perceptions of social expectations (subjective norms), and assessments of behavioral feasibility (perceived behavioral control), thereby influencing their willingness to participate in these volunteer activities.

Therefore, this study posits that the level of awareness of volunteer services that provide sports assistance to people with disabilities can influence willingness to participate through psychological mechanisms. First, the level of awareness is defined as an individual’s cognitive recognition of societal needs or a specific social issue. It refers to an individual’s level of understanding and cognition regarding a matter, serving as a cognitive prerequisite that reflects whether the individual comprehends or acknowledges the existence of certain problems. For example, this includes knowledge of sports-based disability assistance volunteering and recognition of its complexity and urgency. It is not an emotional or evaluative response but rather a purely intrinsic cognitive process that influences an individual’s judgment regarding the necessity of their behavior. This construct provides a specific supplement to the traditional TPB model, offering a critical antecedent that precedes the formation of attitudes, subjective norms, and perceived behavioral control. In this study, awareness serves as a preceding factor before these behavioral beliefs are formed. For example, before participating in sports-based disability assistance volunteering, college students must be aware of the content and factual existence of the volunteer service. Only then can they form the belief that participating in the volunteer service will help alleviate the problem, ultimately developing a positive attitude toward participation in sports-based disability assistance volunteering. Simultaneously, deepening college students’ sense of involvement cultivates positive attitudes toward volunteering in sports-related disability support services, thereby making them more receptive to societal recognition of such volunteerism. This enhances psychological cognition, reinforces subjective norms, and heightens sensitivity to policy directions and social expectations. When college students possess strong intrinsic motivations for participating in sports-based disability assistance volunteer services—such as enthusiasm for teaching, concern for the well-being of people with disabilities, and a sense of personal fulfillment — they are more likely to perceive such involvement as important and worthwhile. This, in turn, enables them to more actively promote and support the participation of people with disabilities in sports activities, thereby gradually amplifying their influence within society.

By continuously leveraging existing resources, implementing behavioral pathways, and reducing perceived barriers, self-efficacy can be enhanced, thereby strengthening individuals’ perceived behavioral control. Behavioral attitudes, subjective norms, and perceived behavioral control collectively function as mediating variables influencing cognitive participation willingness, thereby creating a progressive chain: ‘dispositional evaluation → norm internalization → perceived efficacy.’ As college students’ levels of subjective norms increase, synergistic effects emerge in their expected participation outcomes, responsiveness to social expectations, and confidence in executing actions. This process gradually strengthens their intention to engage in sports-based disability-assistance volunteer services. By incorporating the level of awareness of sports-based disability-assistance volunteer services as an antecedent factor, this model effectively bridges motivation and knowledge, thereby narrowing the gap between knowledge and action. Based on the aforementioned theory, this study proposes the following hypotheses:

*H1*: College students’ level of awareness of volunteer services that provide sports assistance to people with disabilities positively influences their attitudes, subjective norms, and perceived behavioral control.

*H2*: College students’ attitudes, subjective norms, and perceived behavioral control positively influence their willingness to participate in volunteer sports-assistance services for people with disabilities.

*H3*: Attitudes, subjective norms, and perceived behavioral control mediate the relationship between awareness and willingness to participate.

By theoretically augmenting and empirically validating an extended Theory of Planned Behavior that embeds students’ awareness of sports assistance for persons with disabilities, this study provides robust guidance for universities seeking to enhance students’ social responsibility and long-term engagement in volunteer programs — thereby contributing to the continuity of sports-assistance services for people with disabilities.

## 2 Methods

### 2.1 Participants and measurement procedures

Between 20 April and 30 June 2024, an online questionnaire was distributed 697 students majoring in Physical Education and Social Work at one university in Fujian Province, southeastern China. The survey was administered via the online platform WJX platform^1^. Due to resource constraints, convenience sampling was employed for data collection. This study was approved by the Research Ethics Committee of the School of Physical Education and Sport Science, Fujian Normal University. Data collection adhered to the Declaration of Helsinki. At the outset of the survey, introductory instructions explained the study’s purpose, emphasized confidentiality and anonymity, and informed participants of their voluntary participation and right to withdraw at any time. Students interested in volunteering for sports accessibility services were encouraged to participate. A total of 677 valid questionnaires were obtained (677 of 697, response rate = 97.12%). Among the 677 respondents, 350 (51.7%) were male and 327 (48.3%) were female. The sample comprised 211 first-year undergraduates, 286 s-year undergraduates, 94 third-year undergraduates, 35 fourth-year undergraduates, and 51 master’s students.

### 2.2 Research tools

This study used a questionnaire to assess college students’ willingness to participate in sports-based disability-assistance volunteer services. The questionnaire measures students’ understanding of the content of such services and their participation in practical activities. The scale was adapted from the Theory of Planned Behavior (TPB) (Ajzen, 1991) and was modified to create an Expanded Theory of Planned Behavior (ETPB). The traditional Theory of Planned Behavior is organized around four dimensions: attitude, subjective norms, perceived behavioral control, and behavioral intention. Building on this foundation, the present study adds an additional dimension—level of awareness—to form the ETPB model. The questionnaire consists of two parts. Part One collects respondents’ demographic information, frequency of participation in sports-based disability-assistance volunteer services, and perceptions of the current state of such volunteer work (including whether participation should involve spiritual or material rewards). Part Two uses scale items to measure core variables across five dimensions: (1) Behavioral attitude — attitude toward participating in sports-based disability-assistance volunteer services (6 items); (2) Subjective norms (6 items); (3) Perceived behavioral control (6 items); (4) Awareness (3 items); and (5) Willingness to participate (4 items). All items were measured on a 5-point Likert scale. Labels varied by construct: for agreement items 1 = Strongly disagree to 5 = Strongly agree; for familiarity items 1 = Strongly unfamiliar to 5 = Strongly familiar; and for willingness items 1 = Strongly unwilling to 5 = Strongly willing. The questionnaire comprises 25 items across five dimensions (see Table 1 for details).

**Table 1:** Scale design and questionnaire items.

Dimension	Code	Item	Options
Behavioral attitude	BA_1_		
	BA_2_		
	BA_3_		
	BA_4_		
	BA_5_		
	BA_6_		
Subjective norms	SN_1_		
	SN_2_		
	SN_3_		
	SN_4_		
	SN_5_		
	SN_6_		
Perceptual-behavioral control	PBC_1_		
	PBC_2_		
	PBC_3_		
	PBC_4_		
	PBC_5_		
	PBC_6_		
Level of awareness	LA_1_		
	LA_2_		
	LA_3_		
Willingness to participate	WP_1_		
	WP_2_		
	WP_3_		
	WP_4_		
	WP_5_		

### 2.3 Statistical methods

(1) Descriptive statistics and correlational analyses for each variable were conducted using SPSS version 20. To ensure the validity of subsequent analyses, we conducted reliability and validity tests on the scale used in this study to measure college students’ willingness to participate in volunteer sports assistance for people with disabilities. Reliability was assessed using IBM SPSS Statistics, version 20.0. The overall Cronbach’s alpha for the scale was *α* = 0.97, indicating excellent internal consistency.

(2) A structural equation model was estimated using Mplus version 7.4 to examine the relationships among college students’ awareness, attitudes toward sports assistance for people with disabilities, subjective norms, perceived behavioral control, and willingness to participate in volunteer activities. Maximum likelihood estimation with robust standard errors (MLR) was used to estimate model parameters, and bias-corrected nonparametric percentile bootstrap procedures were used to test indirect (mediated) effects. These bootstrap procedures produce bias-corrected 95% confidence intervals for the product of coefficients (indirect effects) and handle non-normal data effectively. An indirect effect was considered statistically significant if its 95% bias-corrected bootstrap confidence interval did not include zero (Wen and Ye, 2014).

(3) To ensure the robustness of the results, we applied specific criteria for reliability and model fit. First, for the measurement model, we assessed reliability using Cronbach’s alpha, with a cutoff of 0.70 (Tavakol and Dennick, 2011). Second, we evaluated overall model fit using multiple indices. Specifically, we considered model fit acceptable if the Comparative Fit Index (CFI) and the Tucker–Lewis Index (TLI) exceeded 0.90 (Hu and Bentler, 1999). Furthermore, we expected the Root Mean Square Error of Approximation (RMSEA) and the Standardized Root Mean Square Residual (SRMR) to be less than 0.08 (Hu and Bentler, 1999). We also reported the chi-square–to–degrees-of-freedom ratio (*χ*^2^/*df*), considering values below 3.0 (and, in some contexts, up to 5.0) indicative of reasonable fit (Hooper et al., 2008).

## 3 Research findings

### 3.1 Reliability and validity testing

To ensure the validity of subsequent analyses, we conducted reliability and validity tests on the scale used in this study to measure college students’ willingness to participate in volunteer sports assistance for people with disabilities. Reliability was assessed using IBM SPSS Statistics, version 20.0. The overall Cronbach’s alpha for the scale was *α* = 0.97, indicating excellent internal consistency. Specifically, the Cronbach’s alpha coefficients for the four subscales were as follows: behavioral attitude (*α* = 0.97), subjective norms (*α* = 0.96), perceived behavioral control (*α* = 0.97), and willingness to participate in volunteer sports assistance for people with disabilities (*α* = 0.96). These results indicate that the subscales and the overall scale exhibit high internal consistency. The CFA results demonstrated a satisfactory model fit. Given the adequate fit, no *post hoc* modifications were deemed necessary based on modification indices. Standardized factor loadings for all items were statistically significant (*p* < 0.001), ranging from 0.80 to 0.96. Composite Reliability (CR) values ranged from 0.95 to 0.98, indicating excellent internal consistency. Furthermore, Average Variance Extracted (AVE) values fell between 0.75 and 0.90, providing evidence of strong convergent validity. Collectively, these findings suggest that the scale possesses robust construct validity.

**Figure 2:**
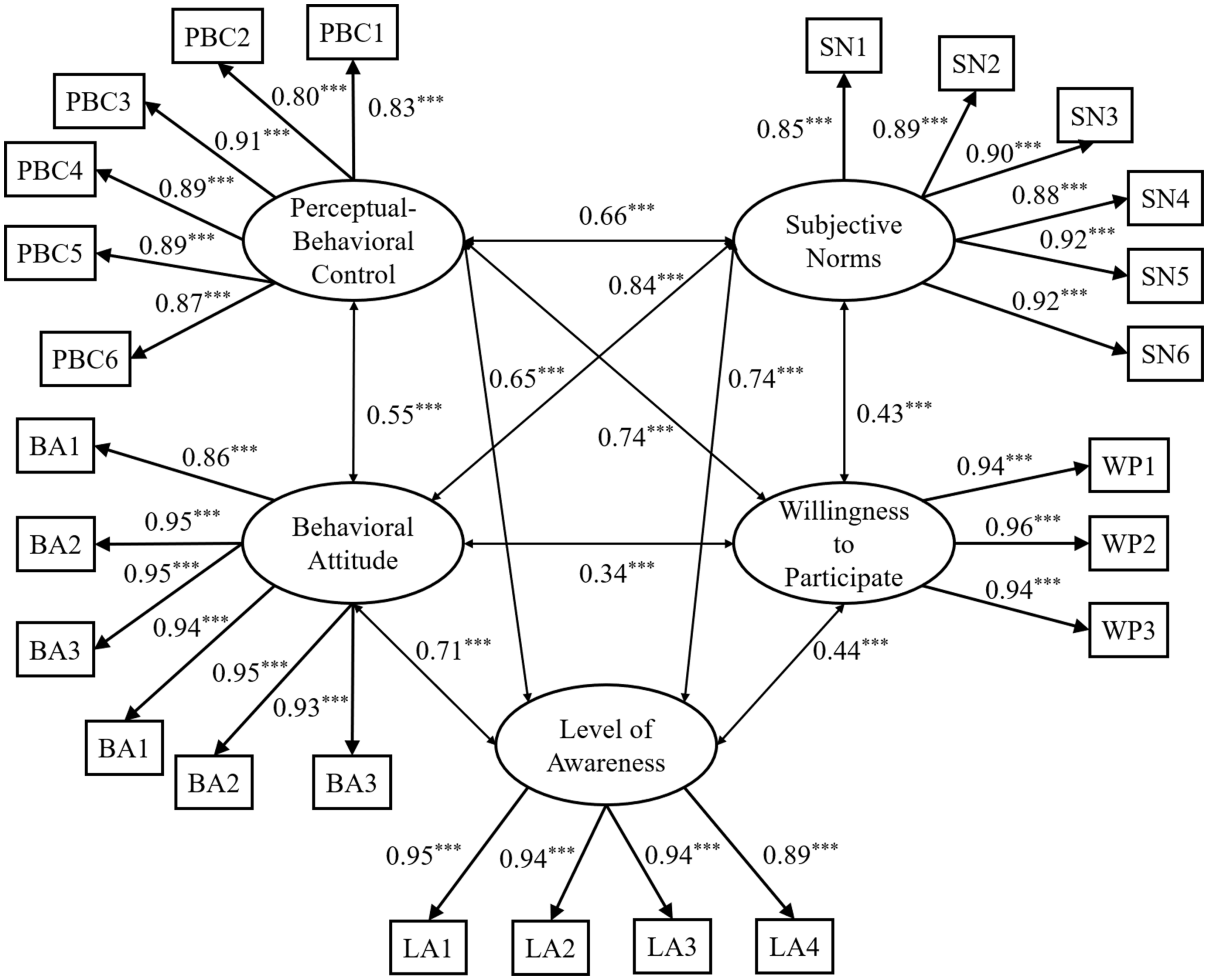
CFA model of the college students’ willingness to participate in sports-based disability assistance volunteer services scale. PBC refers to items from the Perceptual-Behavioral Control subscale; SN refers to items from the Subjective Norms subscale; BA refers to items from the Behavioral Attitude subscale; WP refers to items from the Willingness to Participate subscale; LA refers to items from the Level of Awareness subscale; the numbers in the figure represent standardized results; *** indicates *p* < 0.001.

Confirmatory factor analysis was conducted using Mplus to examine the structural validity of a scale measuring college students’ willingness to participate in sports-assistance volunteer services for people with disabilities. The results indicated that the model fit well (*χ*^2^/*df* = 3.14, RMSEA = 0.06, CFI = 0.95, TLI = 0.94, and SRMR = 0.05). The direct effects model is shown in Figure 2. These results indicate that the scale used in this study demonstrates good construct validity.

The above results indicate that the scale measuring college students’ willingness to participate in sports assistance for the disabled volunteer service used in this study possesses good reliability and validity, and can be further analyzed.

### 3.2 Analysis of structural equation modeling results

#### 3.2.1 Descriptive statistics of each variable

Pearson correlation coefficients were used to assess the relationships among the variables to determine the closeness of the relationships among variables and prepare for the structural equation model. The Pearson correlation analysis (Table 1) indicated that total scores for all subscales were significantly and positively correlated (Table 2). None of the demographic variables, nor the dependent variable — participation willingness — exhibited significant correlations with the primary variables; therefore, they were not included as control variables in subsequent structural equation modeling. The results of the Pearson correlation analysis indicate close relationships among the main variables, and structural equation modeling can be attempted to analyze the data further.

**Table 2:** Descriptive statistics of each variable and the results of their correlation analysis.

Variables	1	2	3	4	5	6	7	8	9
1 Gender^a^	–								
2 Subject category^b^	0.38***	–							
3 Grade	−0.11	−0.19***	–						
4 Number of volunteer service engagements	0.18***	0.32***	−0.07	–					
5 Behavioral attitude	0.05	−0.03	0.02	−0.05	–				
6 Subjective norms	0.07	0.01	0.01	−0.04	0.81***	–			
7 Perceptual-behavioral control	−0.10**	−0.10**	−0.04	−0.17***	0.56***	0.63***	–		
8 Level of awareness	−0.22***	−0.23***	−0.02	−0.23***	0.35***	0.41***	0.71***	–	
9 Willingness to participate	0.07	−0.05	0.01	−0.05	0.69***	0.71***	0.61***	0.43***	–
*M*	0.48	0.52	2.16	4.05	4.18	4.10	3.56	3.08	4.11
SD	0.50	0.50	1.15	1.50	0.89	0.86	0.98	1.17	0.83

^a^Gender is a dummy variable, 0 = female, 1 = male, with the mean representing the proportion of males; ^b^subject Category is a dummy variable, 0 = sports-related majors, 1 = non-sports-related majors, with the mean representing the proportion of non-sports-related majors; *n* = 397; ** indicates *p* < 0.01; *** indicates *p* < 0.001.

**Figure 3:**
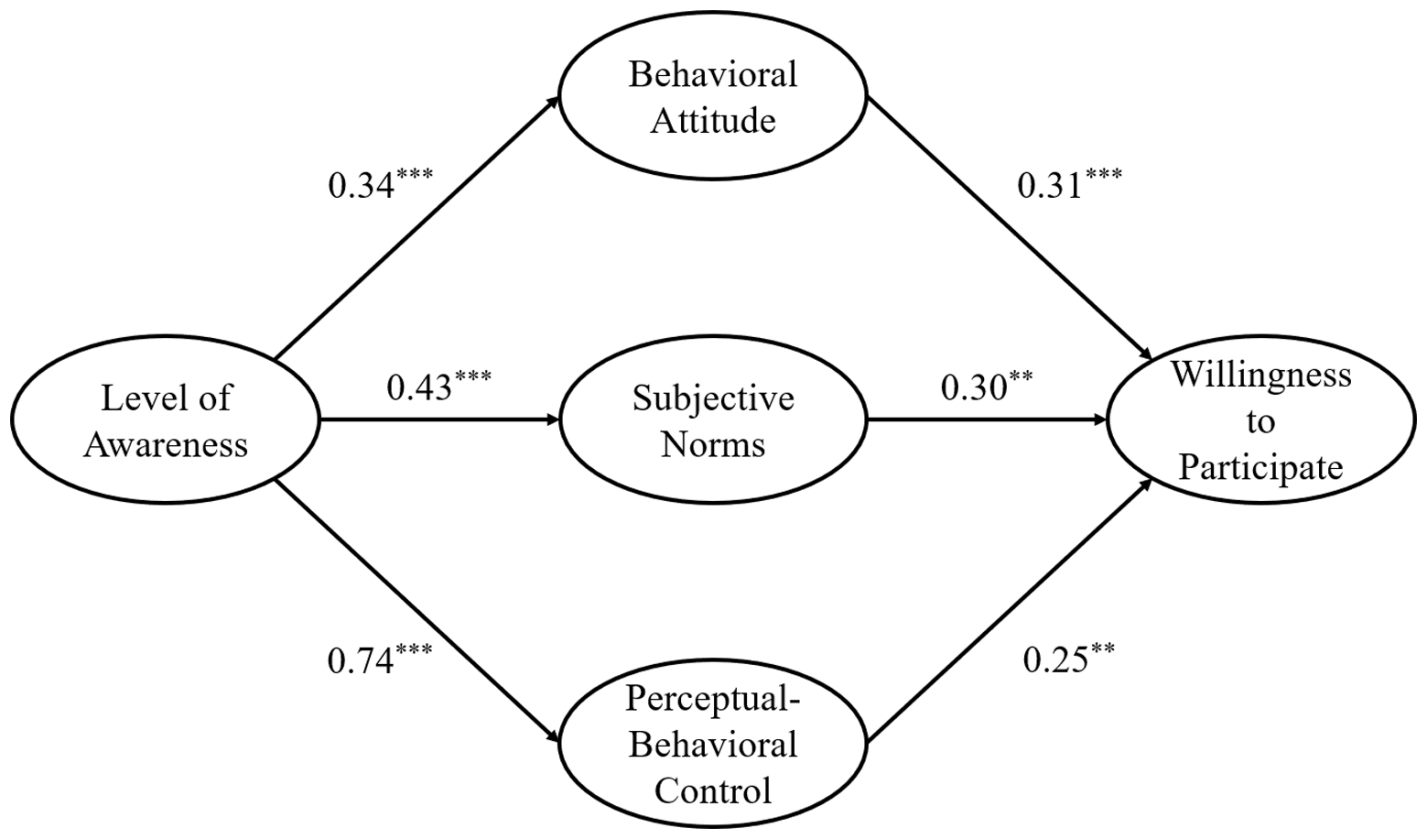
Diagram of SEM model. Figures presented in the figure are standardized solutions; for the sake of brevity, the subject load is not shown in the figure; ** indicates *p* < 0.01, *** indicates *p* < 0.001.

To test hypotheses H1 and H2, we estimated a structural equation model (SEM) using Mplus version 7.4. The structural equation model demonstrated a good fit (*χ*^2^/*df* = 3.14, RMSEA = 0.06, CFI = 0.95, TLI = 0.94, SRMR = 0.05; Figure 3). The results showed that college students’ awareness of volunteer sports-assistance services for people with disabilities significantly and positively predicted their behavioral attitude (*β* = 0.34, *p* < 0.001), subjective norms (*β* = 0.43, *p* < 0.001), and Perceptual-Behavioral Control (*β* = 0.74, *p* < 0.001), thereby validating H1. College students’ behavioral attitude toward sports assistance for people with disabilities significantly influenced their willingness to participate (*β* = 0.31, *p* < 0.001). Subjective norms (*β* = 0.30, *p* < 0.01) and Perceptual-Behavioral Control (*β* = 0.25, *p* < 0.01) also significantly and positively influenced willingness to participate, thus validating H2.

The results indicated that each pathway for this mediating influence was significant and could be used to examine the mediating effect of behavioral attitude, subjective norms and perceptual-behavioral control on the relationship between level of awareness of sports-based volunteer services for people with disabilities and willingness to participate among college students.

### 3.3 Analysis of mediating effect test results

To test H3, the bias-corrected nonparametric percentile bootstrap method (5,000 replicate samples) was used to investigate whether the mediating effect of behavioral attitude, subjective norms and perceptual-behavioral control on the relationship between level of awareness of sports-based volunteer services for people with disabilities and willingness to participate among college students (see Table 2 for the results).

As shown in Table 3, the mediating effect of behavioral attitude, subjective norms and perceptual-behavioral control on the relationship between level of awareness of sports-based volunteer services for people with disabilities and willingness to participate among college students was significant, thus, H3 was verified.

**Table 3:** Mediated effect values and bootstrap test results.

Paths	Effect value	Bootstrap confidence intervals (95%)
Total effect	0.44^***^	[0.38, 0.51]
Level of awareness → behavioral attitude → willingness to participate	0.11^**^	[0.05, 0.18]
Level of awareness → subjective norms → willingness to participate	0.13^**^	[0.5, 0.21]
Level of awareness → perceptual-behavioral control → willingness to participate	0.18^**^	[0.08, 0.29]

The figures listed in the table are standard solutions. ** indicates *p* < 0.01, *** indicates *p* < 0.001.

## 4 Discussion

(1) Previous research has primarily focused on how participation in sports activities can improve the physical health of people with disabilities, enhance their capabilities, and promote social integration (Blauwet and Willick, 2012). The core contribution of this study lies in empirically testing an extended Theory of Planned Behavior (ETPB) model. It confirms that the level of awareness is a key antecedent variable predicting Chinese university students’ intention to volunteer in sports-based disability-assistance services and serves as the primary driver motivating their participation. Adopting an object-centered research perspective, the study positions the selected university students as core participants to explore the role of their subjective cognition and other psychological factors in shaping their willingness to participate. Furthermore, the findings not only validate the traditional TPB constructs but also confirm that the three core variables — behavioral attitude, subjective norms, perceptual-behavioral control — mediate the relationship between the level of awareness and willingness to participate.

Crucially, the study elucidates how the level of awareness fosters stronger social support among university students by shaping more positive behavioral attitude, subjective norms, perceptual-behavioral control. This, in turn, enhances pre-action confidence and ultimately promotes behavioral intention. This study not only validates the applicability of the Theory of Planned Behavior (TPB) to Chinese university students’ participation in sports-based disability-assistance volunteering but also fills a theoretical gap in the literature, offering a new perspective for understanding the complex motivations behind prosocial behavior.

(2) This study found that a heightened level of awareness also provides individuals with the “raw material” for forming attitudes. When college students understand the specific content, social value, and positive changes that sports-based disability volunteer services can bring to people with disabilities, they are more likely to internalize this information, leading to positive emotional evaluations and behavioral tendencies. As students learn more about their volunteer work, they also perceive encouragement and support from teachers, peers, and members of society, thereby gaining positive social pressure. Understanding the volunteer service process and required skills reduces uncertainty, eliminating psychological barriers such as “not knowing how to participate” or “fear of failure.” This, in turn, boosts individual confidence in performing the behavior — a phenomenon observed at the perceived behavioral control level.

(3) Increased levels of awareness directly influence volunteers’ willingness to participate and significantly predict their volunteering intentions. This finding aligns with prior research in the field of environmental behavior, which similarly posits that perceiving a problem effectively serves as a direct driver of behavioral intent (Wang and Mangmeechai, 2021). However, unlike previous studies focusing solely on internal variables within the Theory of Planned Behavior (TPB), this research highlights the unique importance of external information input — specifically, enhanced levels of awareness — as a precursor variable that drives college students’ behavioral attitudes, subjective norms, and perceptual-behavioral control. Particularly among college students, altruistic behavior may be more readily stimulated by new knowledge and changes in cognition. A deep understanding of the specific content and social value of sports-based disability-assistance volunteer activities can also awaken students’ moral norms and their sense of social responsibility. Under certain circumstances, such enhanced awareness may even transcend individual-level behavioral attitudes and perceptual-behavioral control, directly translating into willingness to act.

## 5 Practical implications

(1) Supported by empirical data, this study precisely and effectively expands the traditional Theory of Planned Behavior (TPB), demonstrating the necessity of incorporating a cognitive antecedent variable — level of awareness — into prosocial behavior research and providing new evidence for extending the traditional TPB. First, for institutions such as universities and federations for people with disabilities, the strategies implemented prior to volunteer recruitment are of direct practical significance. Relevant departments should focus on disseminating specific knowledge about, and the social value of, sports-based disability-assistance volunteer services to enhance the level of awareness among volunteers from diverse backgrounds. Second, responsible departments can design differentiated incentive policies tailored to distinct volunteer groups. For students with a high level of awareness, the focus should be on promoting behavioral change and offering corresponding symbolic rewards, such as public recognition or the awarding of digital badges. For students with a low level of awareness, priority should be given to educational outreach campaigns that are combined with tangible incentives. Relevant government departments, disability federations, and other stakeholder organizations can collaborate with universities.

(2) National and local governments should maintain close ties with universities, establish collaborative mechanisms, and deepen the level of awareness of diverse volunteer groups about sports-based disability-assistance volunteer services. Government authorities can leverage local mainstream media and online streaming platforms to broadcast events live and disseminate information online, thereby amplifying the reach of sports-based disability-assistance volunteer initiatives. Organizers should encourage volunteers with prior experience to share their insights and knowledge, thereby raising awareness among peers. For universities, systematically integrating sports-based disability-assistance volunteering into campus-wide general-education elective courses, alongside periodic thematic lectures or seminars, enables students to gain a comprehensive understanding of these services. This approach motivates greater student engagement and ultimately cultivates and expands university volunteer teams.

(3) Research reveals that Chinese university students’ level of awareness regarding sports-based disability-assistance volunteering — including their understanding of relevant policies and volunteer behavior — plays a pivotal role in shaping their behavioral attitude, subjective norms, and perceptual-behavioral control. This awareness serves as the core driver of their willingness to participate. This finding indicates that the provision of abstract, simplistic information is wholly inadequate; only concrete, emotionally resonant knowledge can truly inspire engagement. Therefore, it is recommended that official associations, community organizations, and universities regularly host “volunteer story-sharing sessions.” By presenting authentic case studies from diverse volunteers and their varied insights, these sessions can vividly showcase the content and practical significance of sports-based disability-assistance volunteering. This approach enhances potential participants’ empathy and awareness.

## 6 Conclusion

(1) This study extends the traditional Theory of Planned Behavior (TPB) by proposing an Extended Theory of Planned Behavior (ETPB) that introduces the variable “level of awareness,” thereby providing a more comprehensive explanation of Chinese university students’ intentions to volunteer. The results not only confirm the validity of the traditional TPB constructs but also, more importantly, demonstrate that “level of awareness” serves as an independent, significant predictor of students’ intentions to volunteer.

(2) The ETPB model supports the predictive power of the traditional TPB by showing that behavioral attitude, subjective norms, and perceptual-behavioral control significantly predict university students’ willingness to participate. However, the primary contribution of this study lies in demonstrating that when behavioral attitude, subjective norms, and perceptual-behavioral control are held constant, the level of awareness still continues to exhibit significant and consistent predictive power for university students’ willingness to participate. This indicates that individuals’ level of awareness serves as an independent motivator driving their willingness to participate.

(3) Research indicates that enhancing individuals’ level of awareness regarding sports-based disability assistance, when combined with increased social support, plays a crucial role in boosting college students’ willingness to volunteer for these services. This study contributes to the literature on traditional TPB models by demonstrating that, in domains such as altruism and prosocial behavior, traditional theoretical frameworks alone may be insufficient. Integrating cognitive or moral constructs, such as the level of awareness, into theoretical models can significantly enhance their explanatory power.

## Data Availability

The raw data supporting the conclusions of this article will be made available by the authors, without undue reservation.

## Footnotes

1
https://www.wjx.cn/
